# *DMRcaller*: a versatile R/Bioconductor package for detection and visualization of differentially methylated regions in CpG and non-CpG contexts

**DOI:** 10.1093/nar/gky602

**Published:** 2018-07-09

**Authors:** Marco Catoni, Jonathan MF Tsang, Alessandro P Greco, Nicolae Radu Zabet

**Affiliations:** 1 The Sainsbury Laboratory, University of Cambridge, Cambridge CB2 1LR, UK; 2 DAMTP, University of Cambridge, Cambridge CB3 0WA, UK; 3 School of Biological Sciences, University of Essex, Colchester CO4 3SQ, UK

## Abstract

DNA methylation has been associated with transcriptional repression and detection of differential methylation is important in understanding the underlying causes of differential gene expression. Bisulfite-converted genomic DNA sequencing is the current gold standard in the field for building genome-wide maps at a base pair resolution of DNA methylation. Here we systematically investigate the underlying features of detecting differential DNA methylation in CpG and non-CpG contexts, considering both the case of mammalian systems and plants. In particular, we introduce *DMRcaller*, a highly efficient R/Bioconductor package, which implements several methods to detect differentially methylated regions (DMRs) between two samples. Most importantly, we show that different algorithms are required to compute DMRs and the most appropriate algorithm in each case depends on the sequence context and levels of methylation. Furthermore, we show that *DMRcaller* outperforms other available packages and we propose a new method to select the parameters for this tool and for other available tools. *DMRcaller* is a comprehensive tool for differential methylation analysis which displays high sensitivity and specificity for the detection of DMRs and performs entire genome wide analysis within a few hours.

## INTRODUCTION

DNA methylation is one of the most common epigenetic modifications that is stably inherited and affects gene regulation (1,2). Predominantly, it involves the addition of a methyl group to the carbon-5 position of cytosines and is associated with transcriptional repression. Detection of the methylated cytosines is usually accomplished by bisulfite treatment of the DNA, which leads to unmethylated cytosines being converted to uracil, whilst the 5-methylcytosines remaining unaffected. Given the reduction in cost of DNA sequencing, genome wide bisulfite converted DNA sequencing (BS-seq) has become the method of choice to determine methylation distribution at genomic scale. This approach, despite generating methylation information at single base resolution for theoretically every cytosine in the genome, frequently requires further analysis to efficiently extract methylation information for entire *loci*, or to highlight methylation differences in regions across different conditions, cell types or genotypes.

There are several tools available to detect differentially methylated regions (DMRs) from BS-seq datasets and whilst some of them are implemented in different programming languages (e.g. (3)) or through a web interface, the majority are provided as R packages. Due to its statistical power and the available libraries and packages for bioinformatics analysis, R is the programming language of choice for analysing genomic datasets (4). In particular, Bioconductor (5) represents a collection of R packages aimed to bioinformatics analysis and currently contains more than 1500 packages. The most popular R packages used for detecting differential methylated regions include: *methylKit* (6), *bsseq* (7), *BiSeq* (8), *methylSig* (9), *DSS* (10), *RnBeads* (11), *methylPipe* (12), BEAT (13) and MD3 (14).

Most of these tools were developed for mammalian systems, where DNA is predominantly methylated in CpG context (15–18), and, consequently, they were designed to detect DMRs only in CpG context; e.g. *bsseq* (7), *BiSeq* (8) and *RnBeads* (11). Nevertheless, in plants, non-CpG methylation (in CpHpG and CpHpH contexts, where H can be A, C or T) is also present, playing an important role in epigenetic regulation of transcription (19–23). In addition, recent work has shown the existence of non-CpG methylation in mammalian cell lines (16–18). Whilst there are some tools that can detect differential methylation in a context dependent manner (such as *methylKit, methylSig* and *methylPipe*), they mainly consist of partitioning the genome in tilling bins and pooling together the methylation levels of all cytosines in each bin.

Here, we present a new R package *DMRcaller*, which can compute DMRs between two samples in a methylation context dependent manner. This tool takes as input already pre-processed BS-seq data in the form of methylation calls at each cytosine in the genome. *DMRcaller* implements several methods to detect DMRs and is highly configurable by allowing a wide range of parameters to be controlled. We provide evidence that *DMRcaller* displays the highest accuracy when computing DMRs compared to other available tools, outperforming other methods. Most importantly, we show that the best method to detect DMRs depends on the methylation context (CpG, CpHpG or CpHpH) and *DMRcaller* implements several methods, which makes this package a comprehensive tool for differential methylation analysis. In addition, our results confirm that the detected DMRs are highly reproducible in biological replicates, thus, further providing evidence on the accuracy of the method. Finally, we show that the computing time of DMRs is fast, allowing computing DMRs on whole genome BS-seq datasets generated from human cells/tissues within a few hours.

## MATERIALS AND METHODS

### Description of *DMRcaller*

**Figure 1.**
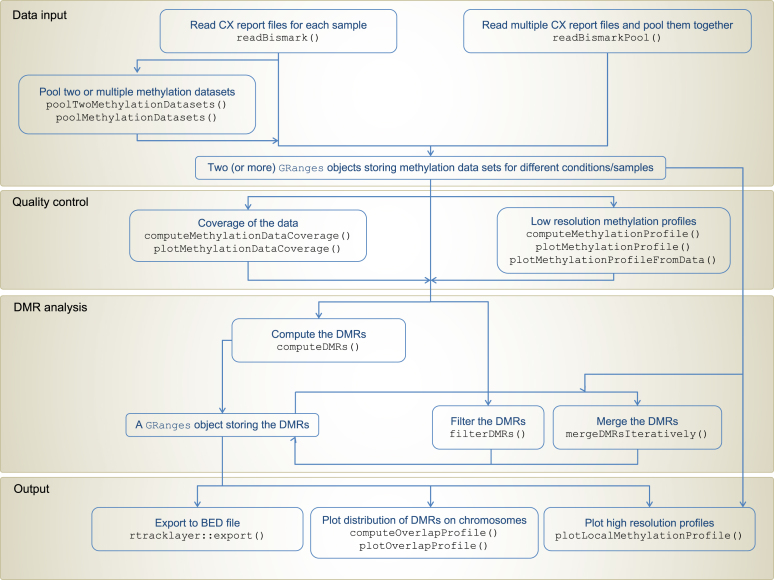
BS-seq analysis workflow using DMRcaller.

We developed an R/Bioconductor (4,5) package called *DMRcaller* (available at http://bioconductor.org/packages/DMRcaller/), which computes DMRs between two conditions. *DMRcaller* uses as input a tab delimited text file with the following columns: chromosome, position, strand, number of reads from methylated DNA, total number of reads, Cytosine context (CG, CHG or CHH) and trinucleotide context (were, instead of H, the exact nucleotide is included). This format is the same of the CX report generated by the popular aligner Bismark (24), however the analysis performed by *DMRcaller* is independent of the aligners that were used as long as the methylation data is formatted accordingly. Figure 1 presents the workflow that one can use to call DMRs using CX report files from two conditions.

#### Data preprocessing

The package computes DMRs with three methods: (i) neighbourhood (*DMRcaller-N*), (ii) bins (*DMRcaller-B*) and (iii) noise filter (*DMRcaller-NF*). The main difference between these methods is the way the methylation data is preprocessed. In the case of the neighbourhood method, the algorithm considers each cytosine independently and, using the raw number of reads and reads from methylated DNA, it calls differentially methylated cytosines (DMCs), which can be extended to DMRs. The second method (*DMRcaller-B*) splits the genome into tilling bins and then pools all reads and reads from methylated DNA in each bin before calling differentially methylated bins. Finally, the noise filter method (*DMRcaller-NF*), preprocess the methylation data by applying a smoothing kernel on the total number of reads and the number of reads from methylated DNA before computing the differential methylation. The noise filter method uses the same assumption of *BSmooth* (7), in particular, that neighbouring cytosines display correlated methylation. *DMRcaller* implements four kernels for noise filtering: (i) uniform, (ii) triangular, (iii) Gaussian and (iv) Epanechnicov; see Supplementary Section S1. For example, the smoothed number of methylated or total reads at a position in the genome is the weighted average over a window (of specified size), where, in the case of a triangular, Gaussian and Epanechnicov kernels, the contribution of distant Cytosines is lower than the contribution of closer ones. As consequence of the smoothing, each position in the genome acquired a smoothed value for both the total number of reads and a number of reads from methylated DNA and, using these corrected values, the function calls the differentially methylated positions (DMPs) using Algorithm 1.

#### Calling DMRs

To detect DMRs, in the case of all methods, the algorithm performs the statistical test for each position, cytosine or bin and then marks as DMRs all positions, cytosines or bins that satisfy the following three conditions:

**Algorithm 1**. *Select DMPs, cytosines, bins or regions*.

- the difference in methylation levels between the two conditions is statistically significant according to the statistical test
- the difference in methylation proportion between the two conditions is higher than a threshold value
- the mean number of reads per cytosine is higher than a threshold

*DMRcaller* implements three statistical tests: (i) Fisher’s exact test, (ii) the Score test (which is a z-test; see Supplementary Section S2 for details) and (iii) Beta regression test for biological replicates. For all statistical tests, we adjust the *P*-values for multiple testing using Benjamini and Hochberg’s method (25) to control the false discovery. Note that first two of these tests lead to similar results, but Score test is slightly faster to compute compared to Fisher’s exact test (see below). Thus, in our analysis, when not computing DMRs on biological replicates, we used Score test to compute differential methylation.

#### Merging DMRs

Adjacent DMPs, cytosines or bins are merged using an iterative process, where neighbouring DMPs, cytosines or bins in the same sequence context (within a certain distance of each other) are joined only if the three conditions listed in Algorithm 1 are still met. These regions are then called DMRs.

Finally, DMRs can be filtered as follows:

**Algorithm 2**. *Filter DMRs*

- Remove DMRs whose lengths are less than a minimum size
- Remove DMRs with fewer cytosines than a threshold value

#### Additional functionality

For a set of potential DMRs (e.g. genes, transposable elements or CpG islands), *DMRcaller* can compute if these regions are differentially methylated by first pooling all reads in each region and then evaluating whether the three conditions in Algorithm 1 are met.

### BS-seq data and processing

We used four previously published BS-seq datasets of *Arabidopsis thaliana* plants: (i) WT replicate 1 (GSM1242401), (ii) WT replicate 2 (GSM980986), (iii) first generation *met1-3* (GSM981031) and (iv) *drm1/2 cmt2/3* (*ddcc*) (GSM1242404) (20,22). These datasets were generated using 3-week-old leaves from *A. thaliana* plants in the Columbia background that were grown under continuous light. In addition, for the biological replicates analysis we used an additional previously published BS-seq dataset of *A. thaliana* plants: (i) WT (GSM2384978) and (ii) first generation *met1-3* (GSM2384980) (26). These datasets were generated using 2-week-old seedlings from *A. thaliana* plants in the Columbia background that were grown under long-day conditions (16 h light, 8 h dark).

Furthermore, we analysed a bisulfite sequencing dataset from rice endosperm (GSM560563) and embryo (GSM560562) together with a list of embryo and endosperm specifically expressed genes published in (27).

Finally, we also used two BS-seq dataset in human IMR90 and H1 cell lines published in (15). In particular, we used a preprocessed version of this dataset from *ListerEtAlBSseq* Bioconductor metadata package (28).

### Computing DMRs

The workflow to compute DMRs is listed in Figure 1. We selected DMRs that contain at least one cytosine, have a size of at least 50 *bp*, display a difference in methylation levels of at least 40% and this difference is statistically significant according to the Score test with an adjusted *P*-value ≤ 0.05. DMRs within a certain distance (equal to twice the window size) of each other were joined if all these conditions were still met. For *methylKit, methylSig* and *methylPipe* we used the set of parameters provided in (12) and only varied the window size in the case of *methylKit* and *methylSig*.

## RESULTS

### Considerations on the methods to detect DMRs

The neighbourhood method assumes the computation of DMCs with no prior filtering or smoothing of the data. Although this is the only implemented method able to call directly single DMCs, one of the disadvantages is that, in the majority of libraries, a consistent subset of cytosines will often have too few reads for the statistical test to be able to call those cytosines as differentially methylated. This is due to the unequal amplification of DNA produced during the library preparation (29), a problem that can be only partially compensated by a higher sequence coverage.

Supplementary Figure S2 shows the coverage of two biological replicates for a BS-seq of *A. thaliana* WT plants. Even for a genome as small as *A. thaliana* (≈130 Mb), only around 60% of the cytosines in CpG context have at least 10 reads in two different BS-seq experiments. In the case of cytosines in CpHpH context, the coverage is even lower than that. This means that DMRs might not be properly detected in a large part of the genome. In another example, a 30× coverage for BS-seq experiment in human cells lead to similar coverage as in the case of *A. thaliana* (60% of the cytosines had at least 10 reads) (15).

One possible solution to alleviate this problem is to smooth the data using a smoothing kernel, which could allow identification of DMRs with much lower coverage (7). The noise filter method uses a moving average to remove non-homogeneous coverage of the genome. The main assumption of the method is that neighbouring cytosines display correlated methylation levels, which seems to be valid in the case of CpG methylation in mammals (30). To determine the usefulness of the noise filter method, we need to test whether this assumption is valid for other organisms (e.g. plants) for both CpG and non-CpG methylation. For this, we considered WT *A. thaliana* plants and computed the Pearson’s correlation coefficient between methylation levels of cytosines in the same context that are separated by a fixed distance. Supplementary Figure S3 confirms that CpG methylation display high correlation within 1 *Kb*, which is similar to the case of CpG methylation in mammalian systems (30). Furthermore, methylation in CpHpG context seems to display similarly high correlation as CpG. In contrast to that, CpHpH methylation displays a low correlation which steeply drops for distances higher than 20 bp, possibly reflecting the different pathway of methylation in this cytosine context in plants (21,23). Together, these results indicate that the noise filter method can be applied on CpG methylation and potentially to CpHpG methylation, but is likely not suitable for CpHpH methylation if used with window sizes higher than 20 bp.

The last method implemented in *DMRcaller* is the bins method ((*DMRcaller-B*). This method partitions the genome in fixed size tilling bins, pooling together all reads in each bin and then performing a statistical test to determine which of the bins display different levels of methylation. Pooling reads within 100 bp bins leads to high number of reads and smaller differences become statistically significant. This is a popular method which is implemented in several other R packages (e.g. (6,9)), but one question which has not been addressed yet is how binning the reads affects the accuracy.

### Comparison with other tools

In Table 1, we listed several R/Bioconductor packages that compute DMRs. In addition to *DMRcaller*, only *methylPipe, methylKit* and *methylSig* can handle non-CpG methylation and, thus, can be applied to plants or to study non-CpG methylation in mammals. It is worthwhile noting that whilst the majority of other packages implement only one method (tilling bins, smoothing, calling differential methylation at individual cytosines, etc.), *DMRcaller* implements three algorithms, thus, allowing more flexibility in applying the most appropriate detection method to call DMR for each cytosine context.

**Table 1.** R/Bioconductor packages for calling DMRs on WGBS

Package	Link	non-CpG	
*DMRcaller*	http://bioconductor.org/packages/DMRcaller/	Yes	
*methylKit*	https://github.com/al2na/methylKit	Yes	(6)
*methylSig*	https://github.com/sartorlab/methylSig	Yes	(9)
*BiSeq*	http://bioconductor.org/packages/BiSeq/	No	(8)
*bsseq*	http://bioconductor.org/packages/bsseq/	No	(7)
*methylPipe*	http://bioconductor.org/packages/methylPipe/	Yes	(12)
*RnBeads*	http://bioconductor.org/packages/RnBeads/	No	(11)
*BEAT*	http://bioconductor.org/packages/BEAT/	No	(13)
*M3D*	http://bioconductor.org/packages/M3D/	No	(14)
*DSS*	http://bioconductor.org/packages/DSS/	No	(10)

**Figure 2.**
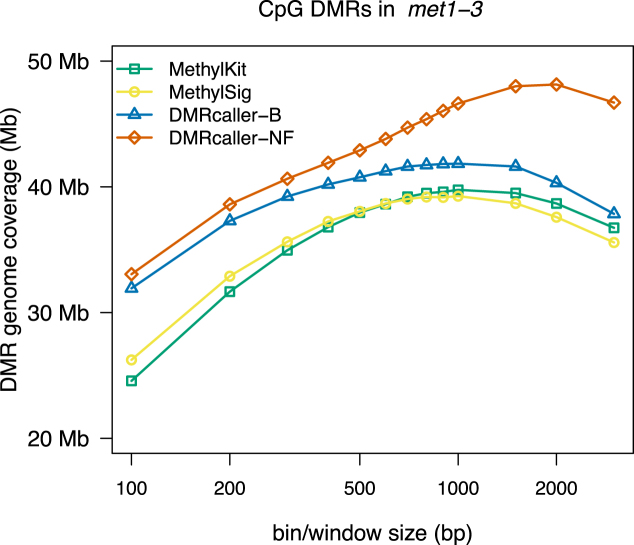
Genome coverage of CpG DMRs between WT and met1-3 *Arabidopsis thaliana* plants as a function of window/bin size. The graph plots the total size of DMRs computed using: (i) *methylKit*, (ii) *methylSig*, (iii) *DMRcaller-B* and (iv) *DMRcaller-NF*.

In our analysis, we compared *DMRcaller* (bins and noise filter methods) with two of the most popular tools for detecting differential methylation: *methylKit* and *methylSig*. To test the performance of our tool to detect DMRs, we first considered the case of CpG methylation in *A. thaliana*. METHYLTRANSFERASE1 (MET1) is the main methyltransferase involved in the maintenance of CpG methylation in *A. thaliana* (26,31,32) and, in *met1-3* mutant, CpG methylation is completely removed; see Supplementary Figure S4A. One parameter that needs to be selected for all four methods is the bin size (window size in the case of *DMRcaller-NF*) and, since all methods lead to DMRs of different size, we computed the DMRs genome coverage (total size of DMRs) for each method to compare the results of the different tools. Taking in account that CpG methylation in *met1-3* mutant is absent, we assume that all DMRs that are detected in the comparison with wild type methylation are true (there is no false positive). Therefore, the method that can recover the highest genome coverage of DMRs will perform best. Figure 2 shows that all tilling bin methods lead to similar DMR genome coverage (39.76 *Mb* for *methylKit*, 39.26 *Mb* for *methylSig* and 41.84 *Mb* for *DMRcaller-B*) with the *DMRcaller-B* displaying a slightly higher value. This can be explained by the fact that, in *DMRcaller*, DMRs are joined if the initial conditions are still met, which will result in larger DMRs. Interestingly, *DMRcaller-NF* produces the highest DMR genome coverage (48.14 Mb), which suggests that this method is the most sensitive of the four methods considered in our analysis.

To investigate the difference between *DMRcaller* and other tools to call DMRs, we compared regions uniquely identified by *DMRcaller-NF* with common regions identified by both *DMRcaller* and *methylKit* (the method that displayed highest DMR genome coverage excluding *DMRcaller*). We observed that DMR portions identified only by *DMRcaller* are mostly adjacent to common regions identified by both methods (10.1 Mb, which represents ≈82% of *DMRcaller-NF* specific DMRs), indicating that most of DNA regions uniquely identified by *DMRcaller* are extension of DMRs called by *methylKit*. These adjacent DMR portions are consistently less methylated in WT samples if compared to common portions (see Supplementary Figure S5B). Considering that CpG methylation in *met1-3* mutant is always absent, this suggests that the smoothing function implemented in the noise filter method can efficiently call regions with a lower methylation change, using information from neighboring higher methylated genomic areas. In contrast, DMRs exclusively called by *DMRcaller-NF* which are not adjacent to commonly identified regions display higher methylation but are shorter in size (see Supplementary Figure S5C), indicating that smoothed data can more efficiently be used to call smaller DMRs, by increasing the amount of informative positions used in the statistical test. Consistently with this, the *DMRcaller-NF* specific regions also display a slightly lower coverage compared to common DMRs (see Supplementary Figure S5A).

Furthermore, we evaluated whether the statistical test used impacted the results. Supplementary Figure S6A and B shows that the there is an almost perfect overlap between the DMRs called in CpG context using the Score test or the Fisher’s exact test and this is valid for both using the noise filter method or the bins method. Nevertheless, the Score test leads to faster computing of DMRs specifically for noise filter method (where the statistical test is performed for each position in the genome); see Supplementary Figure S6C and D.

**Figure 3.**
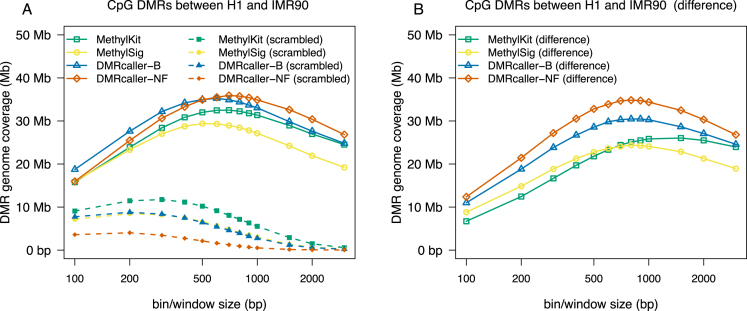
Genome coverage of DMRs between H1 and IMR90 human cells as a function of window/bin size. The graph plots the total size of DMRs computed using: (i) *methylKit*, (ii) *methylSig*, (iii) *DMRcaller-B* and (iv) *DMRcaller-NF*. DMRs on chromosome 1 of the human genome were computed between H1 and IMR90 cells with the four methods. (**A**) Straight lines represent the genome coverage of DMRs on the actual methylation data and dashed lines the genome coverage of DMRs on the scrambled methylation data. (**B**) The lines represent the difference in genome coverage of DMRs between the actual methylation data and scrambled methylation data.

Next, to test the accuracy of *DMRcaller*, we considered a condition where only a few differences in CpG methylation were expected (compare to previous example). For this, we computed the DMRs in CpG context for two BS-seq dataset previously published in (15): (i) IMR90 and (ii) H1 cells. Figure 3A shows genome coverage of DMRs called by the four methods ( *DMRcaller-B, DMRcaller-NF, methylKit* and *methylSig*) on chromosome 1 of the human genome and shows that *methylKit* and *methylSig* display lower genome coverage of DMRs (32.50 and 29.39 Mb) compared to the two *DMRcaller* methods (35.14 and 35.85 Mb). Similar as in the case of CpG methylation in *A. thaliana*, there is an optimal bin/window size that leads to highest genome coverage of DMRs for each method, which corresponds to the highest sensitivity for the methods.

Using the Fisher’s exact test or a Score test could potentially lead to high false positive rates (33–35). Since a good algorithm to call DMRs should be able to discriminate the natural epigenetic variation at level of single cytosine (biological noise) from a consistent stretch of DNA differentially methylated, we generated a scrambled methylation dataset for estimation of false positive call, randomly swapping the methylation values between all cytosines for each methylation context. We used this scrambled dataset to compute DMRs with the same parameters as in the case of the real data for all the methods tested. Due to the fact that the scrambled dataset consists of random methylation data, DNA sequence stretches with consistent differential methylation should appear by chance at a very low rate and the calling algorithm should detect as fewer/smaller DMRs as possible (assuming that all difference found are actually false positive). Moreover, increasing the window/bin size threshold should sharply decrease the genome coverage of DMRs for the scrambled data, whilst should still allow the identification of longer DMRs in the real data.

Figure 3A shows that, for each method, there is a window/bin size that maximises the genome coverage of DMRs in the real dataset whilst displaying low values in the scrambled dataset. Whilst the DMR genome coverage was used as measurement of sensitivity in the analysis of *met1-3* mutant plants, here, we used the difference of the DMR genome coverage calculated for real and scrambled data as estimation of the accuracy in the IMR90 versus H1 comparison. The difference between genome coverage of DMRs in the real dataset and the artificial dataset displays a maximum, indicating that there is a bin/window size that leads to high sensitivity (maximising the genome coverage of DMRs in the real dataset) and at the same time a high accuracy (minimizing the genome coverage of DMRs in the scrambled dataset); see Figure 3B.

Comparing the four methods, we found that *DMRcaller-NF* computes DMRs in CpG context with the highest accuracy among the tested tools (displaying the highest DMR genome coverage in the actual methylation dataset and lowest DMR genome coverage in the scrambled dataset). This is not surprising since CpG methylation highly correlated spatially in both humans (30) and plants (Supplementary Figure S3), and the noise filter algorithm takes advantage of this to increase the significance of the statistical test. Taking this forward, our approach of using scrambled methylation data can be used to compute the window/bin size that will display the highest sensitivity (large genome coverage of DMRs in the real dataset) and at the same time a high accuracy (low-genome coverage of DMRs in the scrambled dataset).

Next, we split the DMRs into regions where there is more methylation in H1 than in IMR90 and regions that display more methylation in IMR90 compared to H1. All methods seem to agree that there is more methylation in H1 than in IMR90 (see Supplementary Figure S7), which is consistent with previous observations (15). Our results show that the majority of CpGs are recovered by all four methods and the overlap between them is between 62 and 78% (Supplementary Figure S7A). Most importantly, between 74 and 78% of the DMRs identified by *methylKit* and *methylSig* are also identified by *DMRcaller-B* and *DMRcaller-NF* (Supplementary Figure S7A).

In our comparison, we did not include *methylPipe*, due to the fact that one cannot call DMRs that contain less than five differentially methylated CpGs and, thus, we could not estimate the accuracy with the scrambled data. Nevertheless, we computed the DMRs with *methylPipe* using the default parameters provided in (12) and compared the results to the ones of the other four methods. Supplementary Figure S7 confirms that *methylPipe* detects DMRs that have a smaller genome coverage (20.9 Mb with higher methylation in H1 cells compared to 35.4 Mb for *DMRcaller-NF*) and the overlap with the DMRs computed by the other methods is usually lower (between 38 and 65%).

**Figure 4.**
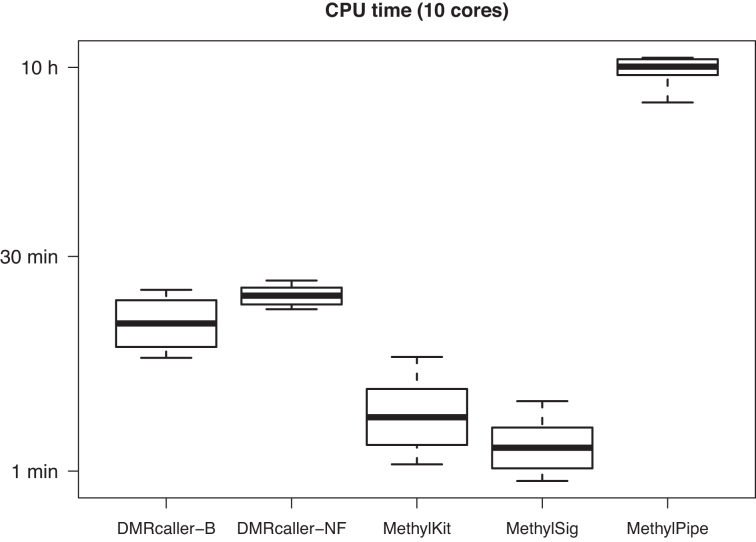
Speed of computing DMRs. The CPU time to compute the DMRs from Figure 3 on methylation data using 10 CPUs on a Mac Pro computer with Intel Xeon E5 2.7GHz 12-core.

Finally, we also compared the speed of these tools. *DMRcaller* (both *DMRcaller-B* and *DMRcaller-NF*) displays slower times compared to *methylKit* and *methylSig*, nevertheless, *DMRcaller* is still fast enough for this not to be an issue; see Figure 4. For example, *DMRcaller* computes the DMRs in less than 30 min on human chromosome 1 (≈ 247 Mb) between H1 and IMR90 cells for window sizes between of 50 and 5000 bp using 10 CPUs. Note that the joining of adjacent DMRs is the cause of the reduce in speed and computing DMRs without merging them leads to similar speeds to to *methylKit* and *methylSig*.

### DMRs in non-CpG context

In plants, methylation in CpHpG context is maintained through a positive feedback loop in between KRYPTONITE (KYP; also known as SUVH4) and CHROMOMETHYLASE 3 (CMT3) or CHROMOMETHYLASE 2 (CMT2) (36–38), whilst methylation in CpHpH context is maintained by the RNA-directed DNA methylation (RdDM) pathway (21,23) . The quadruple mutant *ddcc* (*drm1 drm2 cmt3 cmt2*) leads to complete loss of both CpHpG and CpHpH methylation (22); see Supplementary Figure S4B and C.

**Figure 5.**
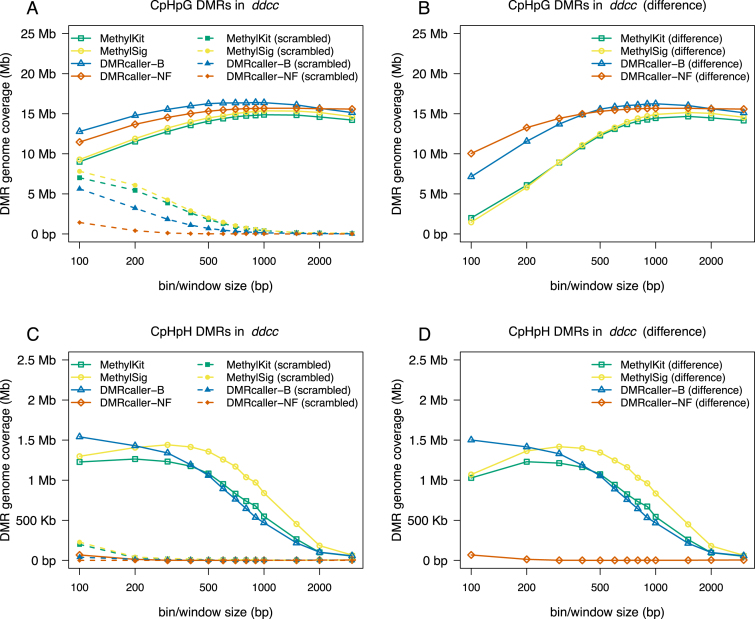
Genome coverage of non-CpG DMRs between WT and ddcc *Arabidopsis thaliana* plants as a function of window/bin size. The graph plots the total size of DMRs computed using: (i) *methylKit*, (ii) *methylSig*, (iii) *DMRcaller-B* and (iv) *DMRcaller-NF*. (**A**) The genome coverage of DMRs in CpHpG context. (**B**) The genome coverage of DMRs in CpHpH context.

Using a similar approach as in the case of CpG methylation, we called DMRs with the four methods for different bin/window sizes. For CpHpG methylation, all four methods lead to very similar genome coverage of DMRs (14.87 Mb for *methylKit*, 15.36 Mb for *methylSig*, 16.41 Mb for *DMRcaller-B* and 15.68 Mb for *DMRcaller-NF*); see Figure 5A. For CpHpH methylation, we found that all tilling bins methods lead to similar results, but the noise filter method is almost unable to detect DMRs; see Figure 5B. This can be explained by the fact that CpHpH, is the only context where the methylation shows very limited spatial correlation; see Supplementary Figure S3.

**Figure 6.**
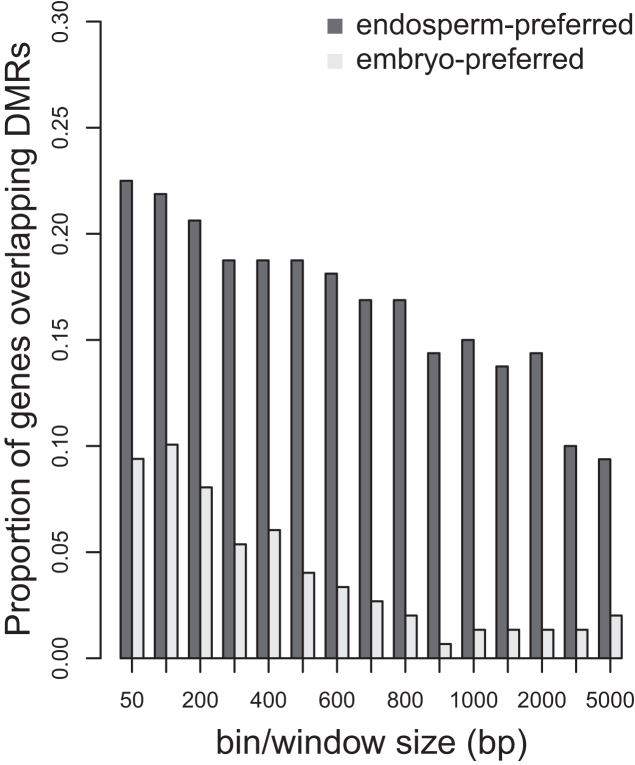
Overlap between CpHpG DMRs called with DMRcaller and preferential expression of genes in rice endosperm or embryo.

Taking together, these results showed that *DMRCaller* is able to call more DMRs compared to other existing tools designed to detect differences in non-CpG context methylation, in conditions where all methylatransferases involved in CpHpG and CpHpH methylation were mutated. However, such extreme condition is rather artificial and not representing a physiological scenario, where epigenetic changes only occur at specific functional regions. Therefore, we used the previously established functional correlation of CpHpG hypomethylation and gene expression observed in rice endosperm to test *DMRcaller* performances in a developmental study (27). In particular, we used the bins method to call DMRs in CpHpG context in rice endosperm compared to embryo from published dataset (27). Then we tested the level of overlap of CpHpG DMRs with a stringent group of 165 genes that displayed a strong preference for endosperm expression, compared with a control group of 153 genes that are preferentially express in the embryo (as defined in (27)). For all windows used, we always observed that DMRs tend to overlap more endosperm-preferred genes (10–22%) compared to embryo-preferred genes (1–10%), providing evidence of functional annotation (see Figure 6). Remarkably, *DMRcaller* outperformed *methylKit* and *methylSig* in both total number of DMRs called and proportion of endosperm-preferred genes overlapping DMRs (see Supplementary Figure S8).

### Computing DMRs from biological replicates

**Figure 7.**
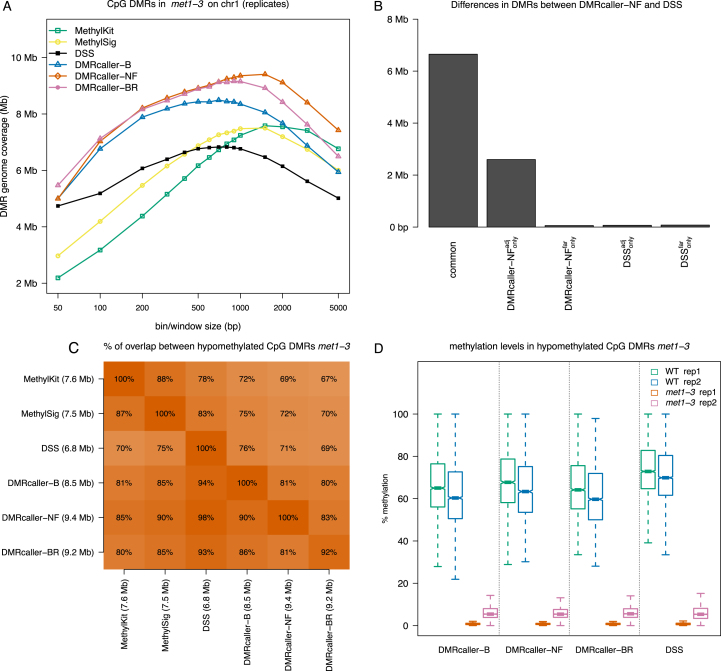
Computing DMRs with biological replicates. We computed DMRs using: (i) *methylKit*, (ii) *methylSig*, (iii) *DSS*, (iv) *DMRcaller* Bins (*DMRcaller-B*), ($v$) *DMRcaller* Noise filter (*DMRcaller-NF*) and (vi) *DMRcaller* with bins and beta regression (*DMRcaller-BR*). DMRs between WT plants and *met1-3* plants were computed on chromosome 1. (**A**) Genome coverage. (**B**) We considered the DMRs identified by *DMRcaller-NF* method and the DMRs identified by *DSS* and split the DMRs into common DMRs, *DMRcaller-NF* specific and *DSS* specific. Furthermore, the DMRs specific to each method were split based on their relative location into: adjacent to common ones ($\textrm{DMRcaller-NF}^{\textrm{adj}}_{\textrm{only}}$ and $\textrm{DSS}^{\textrm{adj}}_{\textrm{only}}$) or far from common ones ($\textrm{DMRcaller-NF}^{\textrm{far}}_{\textrm{only}}$ and $\textrm{DSS}^{\textrm{far}}_{\textrm{only}}$). (**C**) Overlap of DMRs between different methods. (**D**) Methylation level in DMRs for each sample.

To investigate the DMRcaller performance in the detection of DMRs from biological replicates, we considered again the case of WT and *met1-3* mutant *A. thaliana* plants and used one biological replicate from (20) and the second biological replicate from (26). In this analysis we considered *DMRcaller* bins (*DMRcaller-B*) and noise filter (*DMRcaller-NF*) methods and we pooled together the reads from the two replicates. We also used *DMRcaller* and considered explicitly the biological replicates using the implemented beta regression method in conjunction with binning the data (*DMRcaller-BR*) or neighbourhood method (*DMRcaller-NR*); see ‘Materials and Methods’ section. In addition, we used three other methods that model explicitly biological replicates: *methylKit* (6), *methylSig* (9) and *DSS* (10). Due to the long computational time of some of the tools included in this analysis, we detected DMRs only on chromosome 1, using various window sizes. Figure 7A shows that *DMRcaller-NF* detects most DMRs (9.4 Mb) (consistently with what was previously observed), whilst *DSS* detected least DMRs (6.8 Mb); see Figure 3. *DMRcaller-BR* identified a similar number of DMRs as *DMRcaller-NF* (9.2 Mb) and majority of these (≈80%) were detected by all methods; see Figure 7C. When comparing the DMR methylation levels for each sample, we observed that the DMRs detected by *DMRcaller-NF* display similar methylation levels as the ones detected by *DMRcaller-B, DMRcaller-BR* and *DSS*; see Figure 7D.

Finally, we used the neighbourhood method (*DMRcaller-N*) to identify ∼300 000 single cytosines in CpG context that are differentially methylated on chromosome 1 of *met1-3* mutant compared to WT (pooling together reads from replicates) (20,26). Over 81% of these cytosines were included in DMRs called by all methods tested (*DMRcaller-NF, DSS, DMRcaller-BR, DMRcaller-NR*), whilst ∼14% were not included in DMRs by any of these; see Supplementary Figure S9. Both *DMRcaller-NF* and *DMRcaller-NR* detected slightly more DMCs compared to *DSS* and *DMRcaller-BR* (85.6% compared to 81%). This suggests that, in the case tested here, independently of whether we model explicitly biological replicates or pool together reads, the detection of differentially methylated CpGs would lead to similar results.

## DISCUSSION

*DMRcaller* is a simple to use, fast (see Figure 4), powerful and versatile R/Bioconductor package that is able to compute DMRs between two samples. The package implements three methods to compute DMRs: (i) noise filter, (ii) bins and (iii) neighbourhood. The first two methods (noise filter and bins) assume that there is spatial correlation between methylated cytosine (see Supplementary Figure S3) and aim to address low coverage in BS-seq experiments. They achieve this by either smoothing the data with a noise filter (the noise filter method) or binning the data into tilling bins (the bins method). The neighbourhood method can be applied on high coverage dataset and assumes calling individual cytosines as DMCs and joining adjacent DMCs to form longer DMRs.

Most importantly, this package is among few that can compute DMRs in non-CpG context. Non-CpG methylation is an well-established epigenetic mark in plants (19–23) and, recent work, shows that this methylation exists also in some mammalian tissues (16–18). Having the capability to compute DMRs in CpG and non-CpG contexts supports an integrative analysis of DNA methylation and can be useful in investigating the interaction between these types of DNA methylation.

Versions of some of these methods are implemented in several other packages, but, in contrast to *DMRcaller*, these other tools implement only one method. Comparisons of these tools indicate relative success of each method for different datasets, but it seems that none of these methods produce the best results on all datasets. This suggests that the method to detect DMRs should be selected depending on the methylation context, coverage and tissues. Our analysis revealed that, similar to mammals, CpG methylation is spatially correlated in plants and, in addition, non-CpG methylation can also display strong spatial correlation (in the case of CpHpG methylation). Due to this property, we found that the noise filter method has the highest sensitivity (can detect the largest genome coverage of DMRs) in CpG context; see Figures 2 and 3.

Furthermore, we propose a new method to estimate the window/bin size by comparing the genome coverage of DMRs on a dataset where methylation data were scrambled and on the real dataset. To increase sensitivity, we selected values that lead to higher genome coverage of DMRs on the real dataset (to avoid missing differential methylated regions) and, to increase accuracy, we selected values that lead to lower genome coverage of DMRs on the scrambled dataset. Maximizing the difference between there two values would result in a bin/window size that increases sensitivity and accuracy at the same time. Our analysis showed that, for certain window sizes (specific for each methylation context), *DMRcaller* computes a high number of DMRs on the real dataset and a low number of DMRs on the scrambled dataset, which suggests low false negative and false positive rates.

The scrambled dataset permuted the methylation levels to form a null distribution of the methylome profile, from which no difference is expected only if the global methylation level is the same. Thus, we applied the scrambled dataset only for comparison where global methylation levels were similar (75−85%).

Overall, we found that, for both CpG and CpHpG methylation, tilling bins methods and noise filter method will detect DMRs that will cover a large part of the genome (with either *DMRcaller-NF* and *DMRcaller-B* slightly outperforming the other methods); See Figures 2, 3 and 5. Nevertheless, *DMRcaller-NF* call less DMRs in the scramble dataset compared to the other methods. This means that the results of *DMRcaller-NF* are more reliable when the methylation levels are spatially correlated (for CpG and CpHpG context).

In contrast, in the case of CpHpH methylation, there is a reduced spatial correlation of methylation levels and *DMRcaller-NF* is not the appropriate method to detect DMRs. In this case, all tilling bins methods perform well and detect DMRs with high sensitivity and accuracy using bin sizes between 100 and 500 bp. Nevertheless, due to the higher variability of CpHpH methylation, biological replicates would be necessary to detect DMRs with high confidence.

Finally, we show that *DMRcaller* computes DMRs very fast, within similar time scales as other fast methods (*methylKit* and *methylSig*) making the package applicable to larger genomes and that there is a high overlap between biological replicates and *DMRcaller* is able to identify DMRs that recapitulate known biology.

## Supplementary Material

Supplementary Data
