# Supplementary material for: DMRcaller: a versatile R/Bioconductor package for detection and visualization of differentially methylated regions in CpG and non-CpG contexts
Source: Nucleic Acids Res. 2018 Jul 9;46(19):e114. doi: 10.1093/nar/gky602 (PMC6212837; doi:10.1093/nar/gky602)
Supplement: Supplementary Data [file gky602_supplemental_files.pdf]

Supplementary Material to: *DMRcaller*: a versatile R/Bioconductor package for detection and visualization of differentially methylated regions in CpG and non-CpG contexts

S1 Noise filters

DMRcaller implements the following four kernels for noise filtering: (*i*) uniform, (*ii*) triangular, (*iii*) Gaussian and (*iv*) Epanechnicov. If we denote the half of the window size of the Kernel by h, the uniform and triangular kernels can be written as

$$K_{\text{Uniform}}(x) = \frac{1}{2h+1} \tag{S1}$$

$$K_{\text{Triangular}}(x) = \frac{1}{h}\left(1 - \frac{|x|}{h}\right) \tag{S2}$$

The Gaussian and Epanechnikov (quadratic) kernels can be written as

$$K_{\text{Gaussian}}(x, \lambda) = \exp\left(-\lambda x^2\right) \tag{S3}$$

$$K_{\text{Epanechnikov}}(x) = \frac{3}{4}\left(1 - \left(\frac{x}{h}\right)^2\right) \tag{S4}$$

We normalise the Gaussian and Epanechnikov kernels in order for the area under the kernel to add to 1.

$$K_{\text{Gaussian}}(x, \lambda) = \frac{K_{\text{Gaussian}}(x, \lambda)}{\sum K_{\text{Gaussian}}(x, \lambda)} \tag{S5}$$

$$K_{\text{Epanechnikov}}(x) = \frac{K_{\text{Epanechnikov}}(x)}{\sum K_{\text{Epanechnikov}}(x)} \tag{S6}$$

Figure S1 plots the four kernels. In our analysis, we used the triangular kernel, which was previously used to smooth BS-DNAseq data (7).

S2 Statistical tests

DMRcaller uses two statistical tests: (*i*) Fisher's exact test and (*ii*) the Score test. For the Score test, given that m_1 is the number of methylated reads in condition 1, m_2 the number of methylated reads in condition 2, n_1 the total number of reads in condition 1 and n_2 the total number of reads in condition 2, then the Z-score of the Score test is given by:

$$Z = \frac{(p_1 - p_2)\,\nu}{\sqrt{p(1-p)}} \tag{S7}$$

where $p_1 = m_1/n_1$, $p_2 = m_2/n_2$,

$$p = \frac{m_1 + m_2}{n_1 + n_2} \quad \text{and} \quad \nu = \sqrt{\frac{n_1 n_2}{n_1 + n_2}} \tag{S8}$$

The Z-score is then converted to p-value assuming a normal distribution and a two sided test. For the Fisher's exact test, we use R function `fisher.test` from the *stats* package assuming a normal distribution and a two sided test.

S3 Bisultite data analysis

S3.1 Preprocessing

First, we trimmed the reads using Trimmomatic (v0.32) [39] with the following options

```
java -jar trimmomatic-0.32.jar SE -phred33 \
 raw.fasta trimmed.fasta \
 ILLUMINACLIP:adapters.fa:2:30:10 \
 HEADCROP:6 LEADING:3 TRAILING:3 \
 SLIDINGWINDOW:4:15 MINLEN:36
```

where `raw.fasta` is the raw fasta file, `trimmed.fasta` is the trimmed output fasta file and `adapters.fa` contains the list of Illumina adapters.

For methylation call, we use Bismark (v0.14.1) (20) with Bowtie 2 (v2.1.0) [40]. We first prepare the *A. thaliana* genome with Bismark

```
bismark_genome_preparation --verbose \
 --bowtie2 BismarkGenomePreparation/
```

where `BismarkGenomePreparation` directory contains a fasta file with the DNA sequence of the organism studied. After preparing the genome with Bismark, we aligned the reads using Bowtie 2 allowing 3 mismatches

```
bismark --bowtie2 -N 1 -L 20 -p 4 \
 -score_min L,0,-0.6 \
 BismarkGenomePreparation/ \
 trimmed.fasta
```

where `trimmed.fasta` is the trimmed fasta file. Using SAMtools (v0.1.19) [41] we convert the bam files to sam files using the following command

```
samtools view -h \
 -o aligned.sam aligned.bam
```

To remove artefacts from PCR amplification steps during the sequencing protocol we the deduplicate the aligned reads

```
deduplicate_bismark -s aligned.sam
```

Finally, we compute the methylattion levels and generate the CX_report file using the following command

```
bismark_methylation_extractor \
--bedGraph --CX --cytosine_report\
 --genome_folder \
 BismarkGenomePreparation/ \
 aligned_deduplicated.sam
```

References

[39] Bolger, A. M., Lohse, M., and Usadel, B. (2014) Trimmomatic: a flexible trimmer for Illumina sequence data. *Bioinformatics,* **30**(15), 2114–2120.

[40] Langmead, B. and Salzberg, S. L. (2012) Fast gapped-read alignment with Bowtie 2. *Nature Methods,* **9**(4), 357–359.

[41] Li, H., Handsaker, B., Wysoker, A., Fennell, T., Ruan, J., Homer, N., Marth, G., Abecasis, G., Durbin, R., and Subgroup, . G. P. D. P. (2009) The Sequence Alignment/Map format and SAMtools. *Bioinformatics,* **25**(16), 2078–2079.

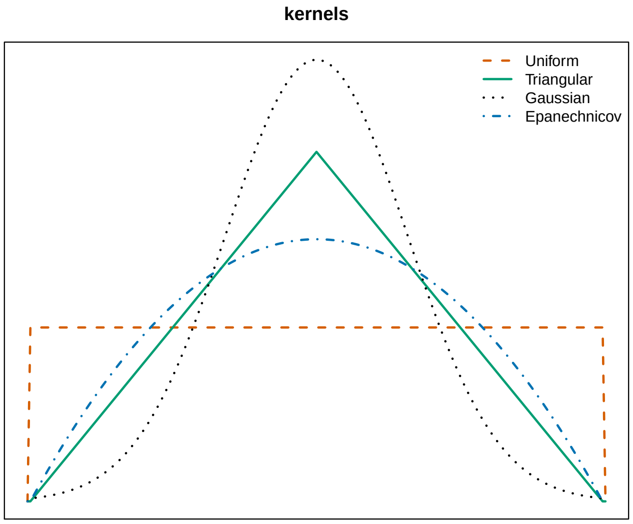

Figure S1: *Smooting kernels*. The noise filter kernels implemented in *DMRcaller*. For all kernels we assumed a window size of 201. For the Gaussian kernel we used a value of $\lambda = 0.0005$.

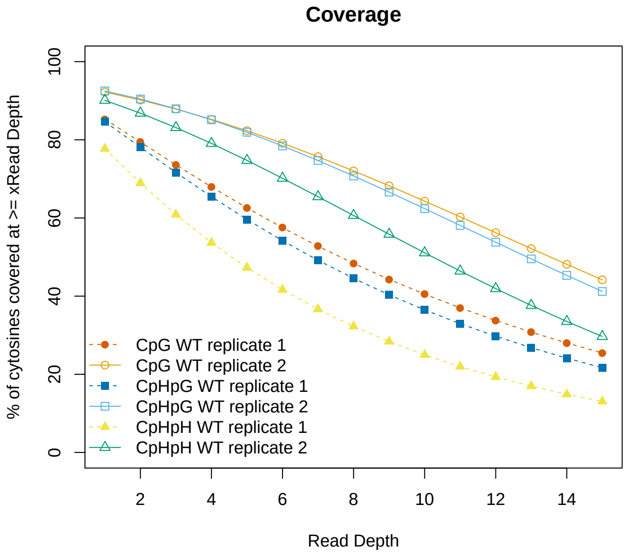

Figure S2: *Coverage of BS-DNAseq datasets in A. thaliana.* The graph plots the percentage of cytosines (in CpG, CpHpG and CpHpH contexts) that have a minim number of reads indicated by the value on the x-axis. We considered BS-DNAseq data from two wild-type *A. thaliana* plants (16,18). Empty symbols indicate data from (16), while filled symbols indicate data from (18).

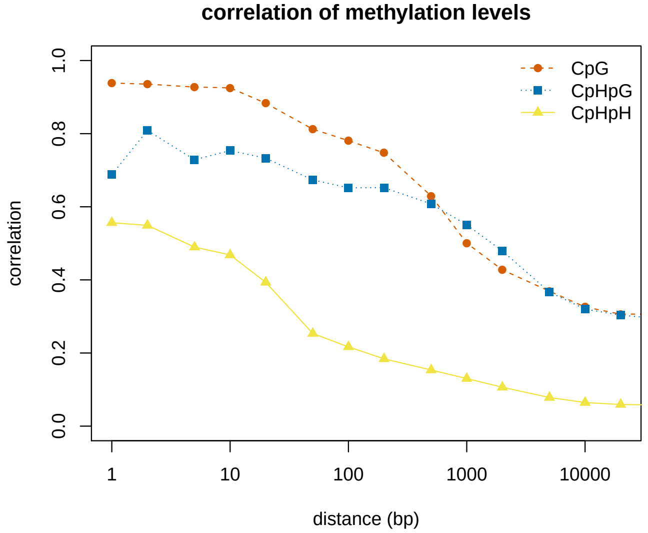

Figure S3: *Correlation between neighbouring cytosines in same context.* The graph plots the correlation between methylation levels of cytosines in a specific context as a function of the distance between the cytosines. We considered BS-DNAseq data from two wild-type *A. thaliana* plants (16,18) and the value plotted is the average of the two replicates. Data for CpG methylation is represented by circles, while data for CpHpG and CpHpH methylation by rectangles and triangles respectively.

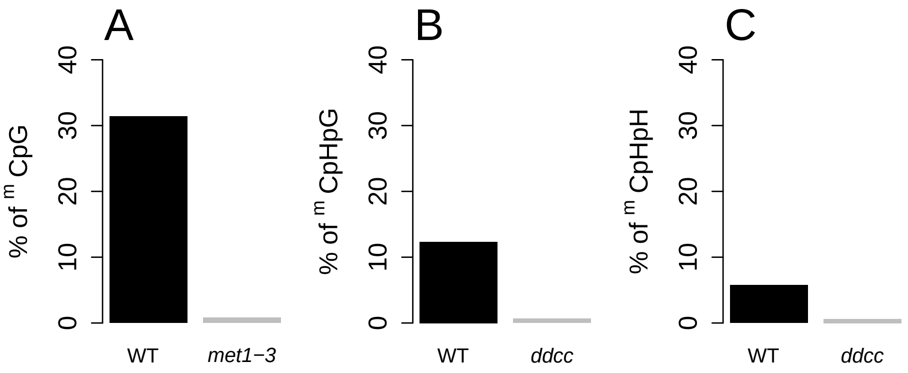

Figure S4: *Methylation levels in WT, met1-3 and ddcc A. thaliana plants.* (*A*) Global CpG methylation levels in WT and *met1-3* plants. (*B*) Global CpHpG methylation levels in WT and *ddcc* plants. (*C*) Global CpHpH methylation levels in WT and *ddcc* plants. We used BS-DNAseq data from (16,18).

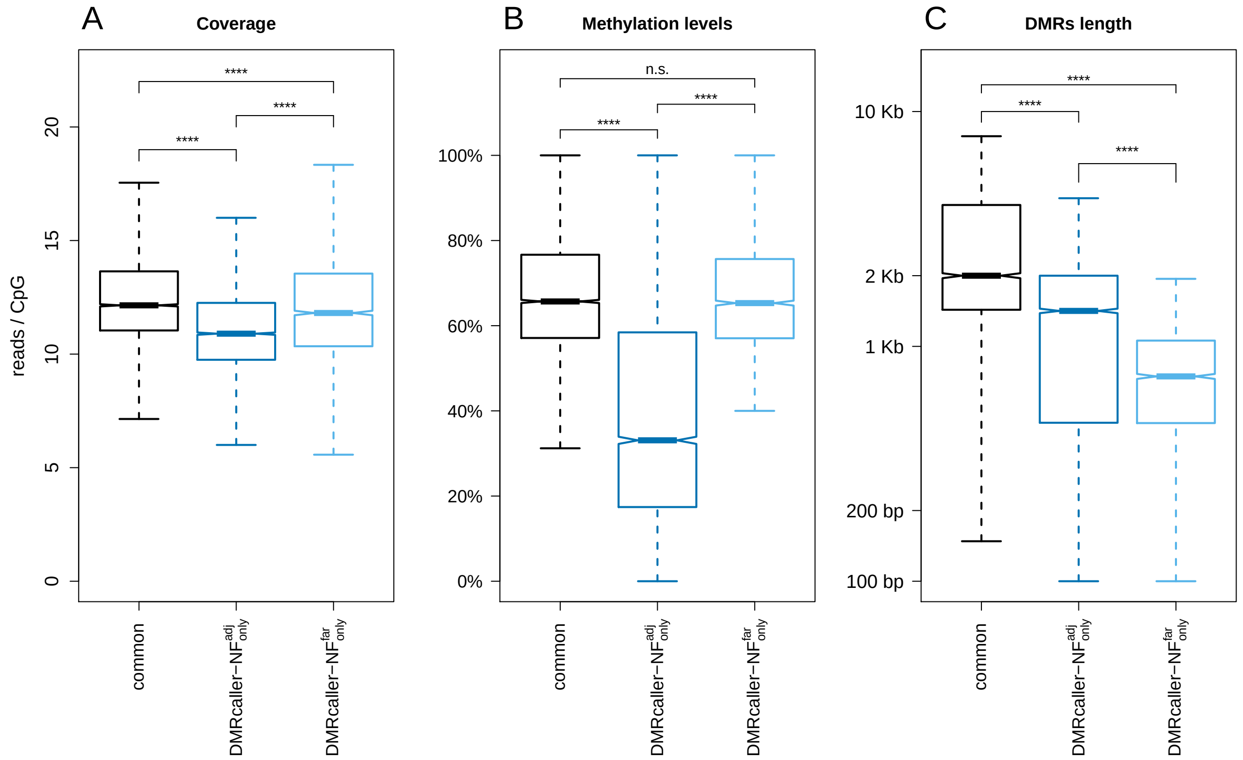

Figure S5: *Characterisation of DMRs identified only by DMRcaller.* We considered the DMRs identified by *DMRcaller* Noise filter (*DMRcaller-NF*) method and the DMRs identified by *methylKit* and split the DMRs into *DMRcaller-NF* specific (12.3 Mb) and common DMRs ($common = 35.8$ Mb). Furthermore, the *DMRcaller-NF* specific were split based on their relative location on the genome into: adjacent to common ones ($\text{DMRcaller-NF}^{\text{adj}}_{\text{only}} = 10.1$ Mb) or far from common ones ($\text{DMRcaller-NF}^{\text{far}}_{\text{only}} = 2.2$ Mb). We plotted: (A) their sequencing coverage (in WT plants), (B) average methylation levels (in WT plants) and (C) DMR length. We performed a Mann-Whitney test and we denoted by $****$ for $p - value \leq 0.0001$ and $n.s.$ for $p - value > 0.05$.

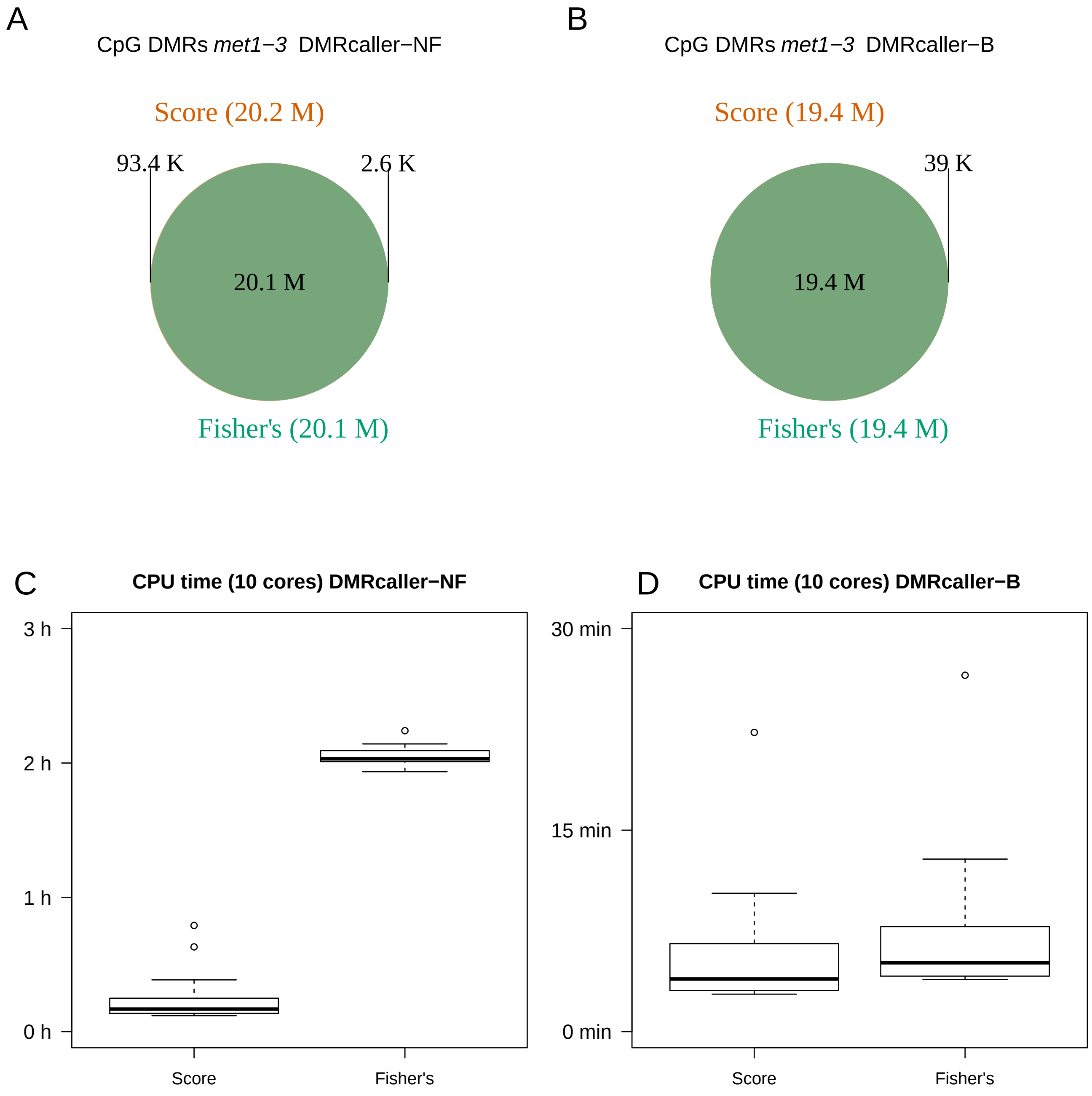

Figure S6: *Comparison of statistical tests.* Comparison of DMRs computed using the Score test and the Fisher's exact test. (A, B) Overlap of DMRs and (C, D) CPU time for DMRs computed with *DMRcaller-NF* method (A, C) and *DMRcaller-B* method (B, D). (A, C) Hypomethylated DMRs in CpG context (with lower methylation in *met1-3* plants compared to WT) were computed using the noise filter approach and a window size of 2000 *bp*; see Figure 2. (B, D) Hypomethylated DMRs in CpG context (with lower methylation in *met1-3* plants compared to WT) were computed using the bins approach assuming a window size of 900 *bp*; see Figure 2. The computations were performed using 10 CPUs on a Mac with Pro Intel Xeon E5 2.7GHz 12-core.

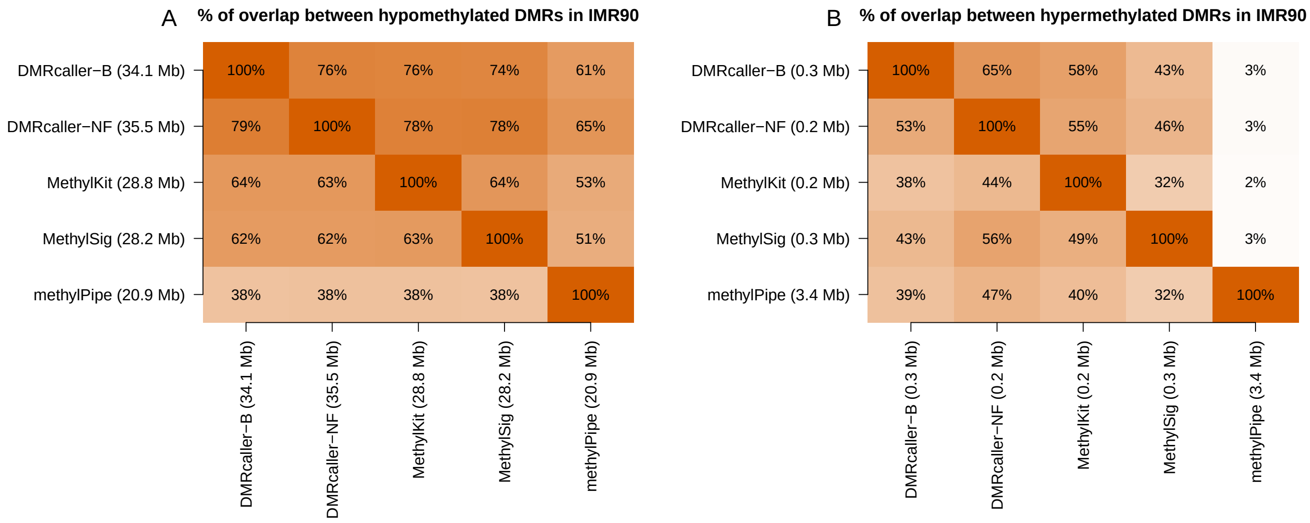

Figure S7: *Overlap of DMRs between H1 and IMR90 human cells computed with different methods.* The plot represents the overlap of the DMRs computed with: (*i*) *DMRcaller* Bins (*DMRcaller-B*), (*ii*) *DMRcaller* Noise filter (*DMRcaller-NF*), (*iii*) *methylKit*, (*iv*) *methylSig* and (*v*) *methylPipe*. The number in the parentheses of each label on the axes represents the genome coverage of DMRs called with the corresponding method. The window/bin size was selected to maximise the difference between the genome coverage of DMRs computed on the real data and on the scrambled data (800 *bp* for *DMRcaller-B*, 800 *bp* for *DMRcaller-NF*, 1500 *bp* for *methylKit* and 800 *bp* for *methylSig*). The number indicate the percentage of DMRs computed with the method on the x-axis that overlap with DMRs computed with the method on the y-axis.

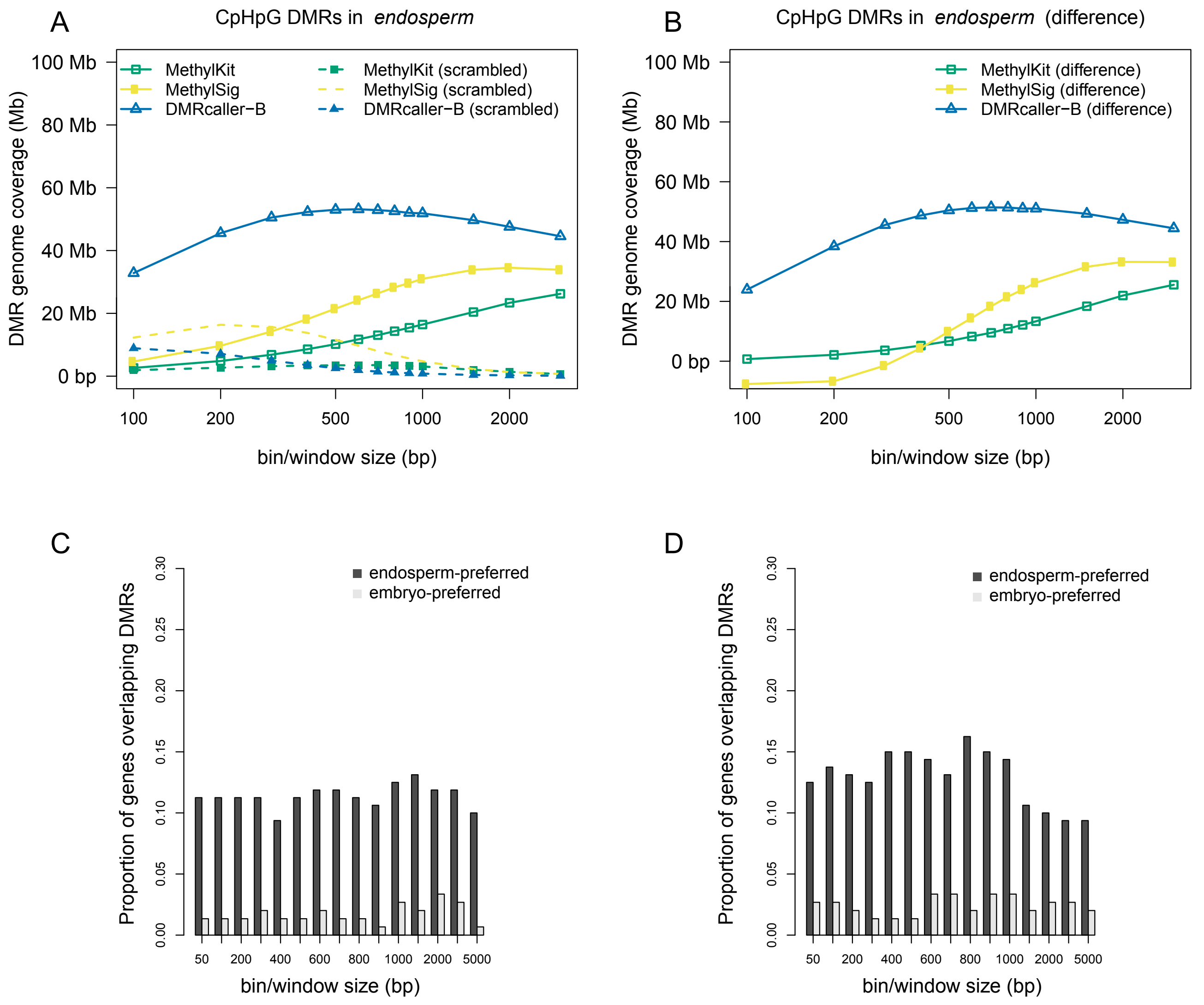

Figure S8: *CpHpG DMRs in rice endosperm.* The graph plots the total size of DMRs computed using: (*i*) *methylKit* , (*ii*) *methylSig* and (*iii*) *DMRcaller-B*. DMRs between endosperm and embryo in rice were computed. (*A*) Straight lines represent the genome coverage of DMRs on the actual methylation data and dashed lines the genome coverage of DMRs on the scrambled methylation data. (*B*) The lines represent the difference in genome coverage of DMRs between the actual methylation data and scrambled methylation data. (*C*) Overlap between CpHpH DMRs called with *methylKit* and gene loci preferential expressed in rice endosperm or embryo. (*D*) Overlap between CpHpH DMRs called with *methylSig* and gene loci preferential expressed in rice endosperm or embryo.

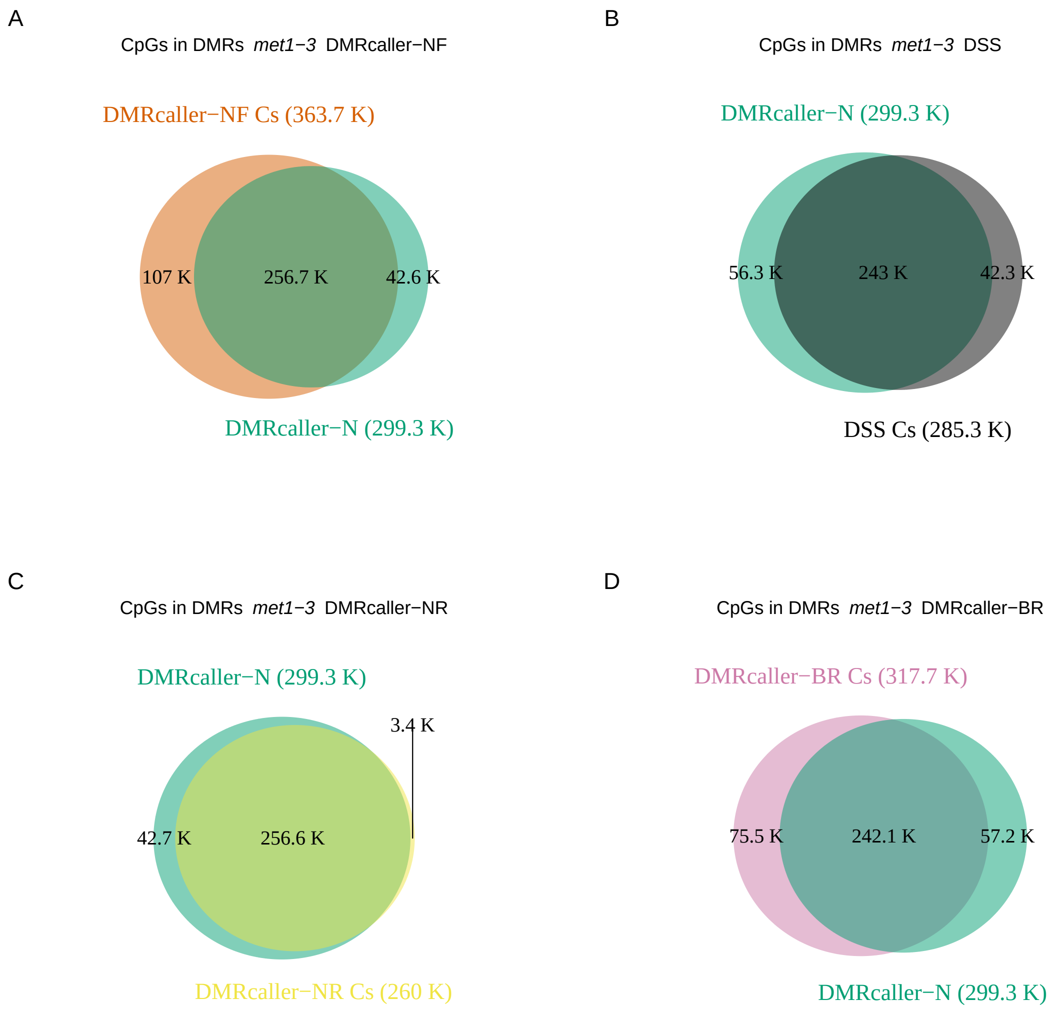

Figure S9: *Differentially methylated Cytosines.* Comparison of CpGs that are differentially methylated computed using the *DMRcaller* Neighbourhood method (*DMRcaller-N*). (*A*) comparison between *DMRcaller-N* and *DMRcaller-NF*. (*B*) comparison between *DMRcaller-N* and *DSS* (*C*) comparison between *DMRcaller-N* and *DMRcaller* with neighbourhood method and biological replicates (*DMRcaller-NR*). (*D*) comparison between *DMRcaller-N* and *DMRcaller* with bins method and biological replicates (*DMRcaller-BR*).
